# Smoother: on-the-fly processing of interactome data using prefix sums

**DOI:** 10.1093/nar/gkae008

**Published:** 2024-01-28

**Authors:** Markus R Schmidt, Anna Barcons-Simon, Claudia Rabuffo, T Nicolai Siegel

**Affiliations:** Division of Experimental Parasitology, Faculty of Veterinary Medicine, Ludwig-Maximilians-Universität München, Munich, Germany; Biomedical Center, Division of Physiological Chemistry, Faculty of Medicine, Ludwig-Maximilians-Universität München, Munich, Germany; Division of Experimental Parasitology, Faculty of Veterinary Medicine, Ludwig-Maximilians-Universität München, Munich, Germany; Biomedical Center, Division of Physiological Chemistry, Faculty of Medicine, Ludwig-Maximilians-Universität München, Munich, Germany; Division of Experimental Parasitology, Faculty of Veterinary Medicine, Ludwig-Maximilians-Universität München, Munich, Germany; Biomedical Center, Division of Physiological Chemistry, Faculty of Medicine, Ludwig-Maximilians-Universität München, Munich, Germany; Division of Experimental Parasitology, Faculty of Veterinary Medicine, Ludwig-Maximilians-Universität München, Munich, Germany; Biomedical Center, Division of Physiological Chemistry, Faculty of Medicine, Ludwig-Maximilians-Universität München, Munich, Germany

## Abstract

Nucleic acid interactome data, such as chromosome conformation capture data and RNA–DNA interactome data, are currently analyzed via pipelines that must be rerun for each new parameter set. A more dynamic approach is desirable since the optimal parameter set is commonly unknown ahead of time and rerunning pipelines is a time-consuming process. We have developed an approach fast enough to process interactome data on-the-fly using a sparse prefix sum index. With this index, we created Smoother, a flexible, multifeatured visualization and analysis tool that allows interactive filtering, e.g. by mapping quality, almost instant comparisons between different normalization approaches, e.g. iterative correction, and ploidy correction. Further, Smoother can overlay other sequencing data or genomic annotations, compare different samples, and perform virtual 4C analysis. Smoother permits a novel way to interact with and explore interactome data, fostering comprehensive, high-quality data analysis. Smoother is available at https://github.com/Siegel-Lab/BioSmoother under the MIT license.

## Graphical Abstract

**Graphical Abstract:**
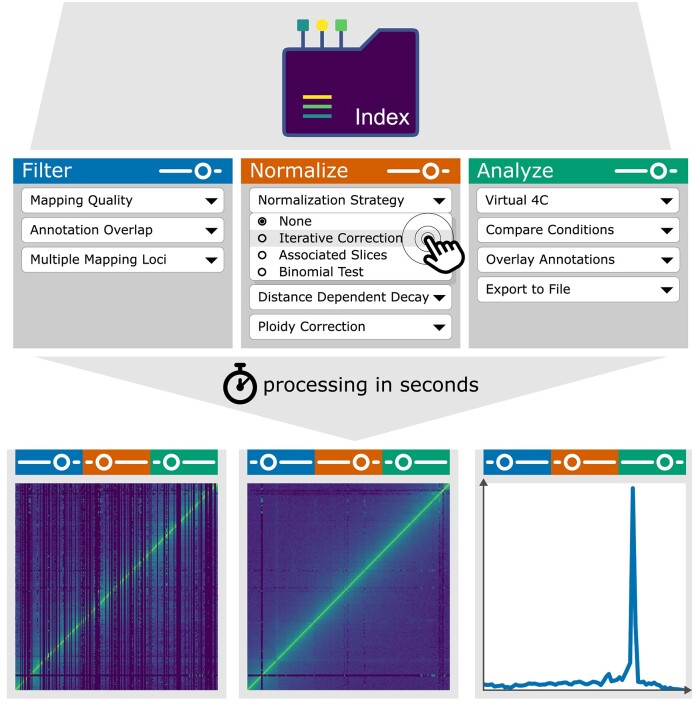

## Introduction

Chromosome conformation capture (3C) methods, RNA–DNA and RNA–RNA interactome capture methods sequence pairs of nucleic acid fragments that are in spatial proximity. 3C methods include Hi-C (1), Micro-C (2), RCMC (3), Pore-C (4) and SPRITE (5), while methods like RADICL-seq (6), GRID-seq (7), RD-SPRITE (8) or RIC-seq (9) are commonly used to capture the RNA interactome. These sequenced fragment pairs are called ‘interactions’ and one fragment of the pair is an ‘interaction partner’. Population-level sequencing of such interactions, hence, reveals the average contact frequencies of genomic loci. Such contact frequencies are commonly processed into two-dimensional heatmaps by (i) mapping, (ii) filtering, (iii) binning, (iv) normalizing and (v) displaying (10–13): (i) An aligner is used to find one or many mapping loci for each interaction partner. Each locus is assigned a mapping quality, which expresses the aligner's confidence in the correctness of the locus. (ii) Unwanted interactions, such as those with low mapping qualities or those mapping to multiple loci are filtered out. (iii) Interactions are placed into bins of a given resolution. From this step on, the number of interactions within a bin is typically stored in favor of individual interactions. (iv) Subsequently, normalization strategies are applied to correct for different biases occurring during sample preparation or data processing, e.g. during PCR amplification, mapping or filtering. The normalization of choice depends on the data type and the analysis to be performed. Commonly, 3C data are normalized with Iterative Correction (IC) (12) or KR-balancing (13), while Binomial test (6) and Associated Slices normalization (7) are used for RNA–DNA interactome data. IC and KR-balancing equalize the visibility of a matrix by equalizing its column and row sums, while the Binomial test determines the statistical significance of each bin over the genome-wide coverage of the interacting RNA. Associated Slices normalizes each bin by the sum of *trans*, chromatin-associated interactions that share the bin's interacting RNA. (v) Normalized data are displayed as two-dimensional heatmaps either by conversion into images or inspection with interactive viewers (14–18). These viewers aggregate heatmaps of various resolutions into a single file to be able to display both high-resolution images for small subsections of the dataset and low-resolution images for overviews of the dataset. However, parameters such as the available resolutions, the type of normalization, or filter thresholds (mapping quality, annotation overlap) remain fixed at this point.

In many cases, nucleic acid interactome data are used in an exploratory fashion, e.g. to gain a general overview of the DNA-DNA or RNA–DNA interactome. Even if there is a specific hypothesis, e.g. that the depletion of a protein will lead to a decrease in interactions between two specific genomic loci, optimal parameters for data analysis are commonly unknown in advance. Yet, pipeline-based approaches require the commitment to specific parameter values from the outset. This routinely requires the rerunning of pipelines until a suitable parameter set has been found. With runtimes of up to several hours per step, such pipeline rerunning is a time-consuming process.

To address this challenge, we created Smoother, an interactome data analysis tool that allows changing analysis parameters by interaction with a graphical user interface. Here, the effects of these parameter changes are displayed as an updated heatmap within seconds. Interactive changes can be made for any of the filtering, binning, normalizing, and displaying steps. For example, reads with low mapping quality can be removed, the normalization strategy can be changed entirely or run with different parameters, and bin size can be modified. A naïve strategy to offer such interactivity would be to rerun the pipeline each time a parameter changes. However, such rerunning is not practicable, due to the large amount of time required to process interactome datasets. Alternatively, to eliminate the need of having to reprocess the datasets continuously, heatmaps for all possible parameter permutations could be precomputed. While such precomputed heatmaps could be displayed very efficiently, the large number of parameter permutations makes it impossible to precompute and store all possible heatmaps for a given dataset. To solve this problem, we developed a strategy to precompute and store an encoded and compressed version of the heatmaps for all possible parameter permutations using prefix sums (17,18).

## Materials and methods

### Using prefix sums for counting the number of interactions in a bin

With the large size of interactome datasets of >${10}^7$ interactions per replicate, it was crucial to make the runtime of our approach independent of the dataset size. The same applies to large genome sizes (*T. brucei*’s genome assembly is 50 Mb long). With such large genomes, gaining an overview of the entire dataset requires computing large bins. Hence, no matter the number of interactions, the bin size and the genome size, processing a heatmap should always take the same amount of time.

Such constant heatmap processing time can be achieved using prefix sums (17,18): We first introduce the one-dimensional case with an example. Let us consider a genome of size 8 and a set of interactions at the positions $\{ {1,\ 3,\ 3,\ 7} \}$. We want to efficiently count the number of interactions in any given bin. For example, a bin with a range from 2 to 6 contains 2 interactions. To do so, we first compute the prefix sums for all positions on the genome. The prefix sum of a position is the number of interactions at or before this position. In our example, the prefix sum at position 6 would be 3, since 3 interactions (1, 3 and 3) lie at or before position 6. We compute these prefix sums once and store them in a file. Next, we use these prefix sums for counting the number of interactions in bins. This is done by subtracting the prefix sum for the start position of the bin from the prefix sum of its end position (e.g. for the bin starting at 2 and ending at 6: $3 - 1 = 2$). Such subtraction yields the correct number of points since the prefix sum for the end position holds the number of interactions that lie at or before the end of the bin, while the prefix sum for the start position holds the number of interactions that lie at or before the start of the bin. Subtracting both numbers leaves us with the number of interactions within the bin. As intended, the effort required for counting the number of interactions in any bin using prefix sums is independent of the number of interactions, the genome size, and the bin size: for any bin and dataset, two prefix sums are looked up and subtracted. Supplementary Note S1 generalizes this approach to the $d$-dimensional case.

Alternative data structures to prefix sums could be R-trees or range trees. We opted to use prefix sums as they offer the fastest querying times by orders of magnitudes. Supplementary Note S2 compares these three data structures in more detail.

### Filtering interactions by mapping quality using an additional dimension

Mapping quality expresses the aligner's confidence in the correctness of the alignment of a read. Therefore, a read that aligns equally well to two positions of the reference genome would produce two alignments with mapping quality zero, while a read that is completely distinct from all but one genomic locus would yield one alignment with a very high mapping quality (19).

To filter out interactions with low mapping qualities on-the-fly, we moved from 2D to 3D prefix sums. Here, each interaction still has an $x$ and $y$ position based on the alignments of the interaction partners. Additionally, we give each interaction a *z* position according to the alignments’ mapping qualities (we use the lower of the two mapping qualities). Then, instead of querying one rectangle for each bin of the heatmap, we query a cube. By varying the *z* position of the bottom face of these cubes, we filter out interactions with low mapping qualities (Figure 2A).

A $d$-dimensional index requires ${2}^d$ lookups per counting operation, where each lookup corresponds to one corner of the queried $d$-hyperrectangle (Supplementary Note S1). Hence, introducing a new dimension would slow our approach down by a factor of two. However, we know that the prefix sum of a position smaller than all interactions must be zero. Thus, points of a queried $d$-hyperrectangle that lie below all interactions need no lookup, possibly reducing the query complexity. To make use of this reduced query time, we invert the $z$-dimension of our index. This way, interactions with high mapping qualities lie at the bottom, while those with low mapping qualities are positioned at the top. We then filter out low mapping qualities by varying the height of the queried cubes’ top face (instead of the bottom face). Importantly, this way, the bottom face of all cubes is always located below the highest possible mapping quality. Hence, we know that all four corners of the bottom face have a prefix sum of zero and require no lookup. This reduces the number of lookups to four (instead of the eight usually required for cubes), making this filter have no query time penalty whatsoever.

### Rescuing multimapping interactions by counting rectangles instead of points

As described above, we can use an additional dimension to filter out interactions that align equally well to multiple positions of the genome by filtering out interactions with mapping quality zero. However, we also offer a complementing feature to deal with such ambiguous alignments: an interaction that maps to multiple positions can lead to two separate situations, where either (i) alignments are distributed over several bins or (ii) all alignments are within the same bin. While displaying distributed alignments (case i) would lead to more noise in the heatmap, displaying confined alignments (case ii) does not. Case ii does not lead to noise, since, while we do not know the exact loci of a confined interaction, we do know that all possible loci of the interaction are within the same bin. Hence, we can safely count this interaction towards the bin. This is a simplified implementation of the approach of Zheng *et al.* (20).

For implementing this feature, and so distinguishing cases (i) and (ii), we extended the prefix sum approach to storing and counting rectangles instead of points. These rectangles are generated by first determining all alignment loci of an interaction and then storing the smallest rectangle that encloses all these loci using prefix sums. We next filter out all distributed alignments (case i), by only counting rectangles that are fully enclosed by a bin (Figure 2B).

First, we introduce the one-dimensional case, where fully enclosed intervals are counted (instead of rectangles): There, we count how many multimapper-intervals are fully enclosed in a given bin by subtracting the number of multimapper-intervals starting before the bin from the number of multimapper-intervals ending after the bin (see Supplementary Note S3). This filters our intervals that overlap either edge of the bin. However, the approach cannot deal with intervals that fully enclose the bin (see Supplementary Note S3). For filtering out such enclosing intervals we introduce an additional dimension. In this dimension intervals are placed at a position according to their width. We then filter out all intervals larger than the bin, and so any intervals that could potentially fully enclose the bin, by adjusting the bounds of the queried region in the filter dimension. For this additional dimension, no inversion of the axis is needed to improve runtime (as opposed to the mapping quality dimension), as we filter out large values to begin with. Supplementary Note S3 details how this approach can be generalized to the $d$-dimensional case, storing *d*-dimensional hyperrectangles. Further, Supplementary Note S3 details how to count all overlapping instead of enclosed hyperrectangles.

### Filtering by annotation using two additional dimensions per annotation type

RNA–DNA interactome data is often analyzed considering merely reads that originate from gene bodies. Since our goal is to make our index agnostic to the type of data that is stored, all analysis-related parameters shall be set during querying. Hence, we require a query time option to filter out reads that do not overlap gene bodies, or, more broadly, any chosen type of genomic annotation. For this, we introduced two filter dimensions per annotation type, where one dimension stores x- and the other y-axis overlaps. Interactions are then placed at position zero or one in each of these dimensions depending on whether they overlay the annotation type or not.

Annotation overlap is checked individually for each locus of a multimapping interaction. The multimapper is then considered to overlap any annotation that at least one of its mapping loci overlaps.

### Annotation coordinates

Some RNA interactome analyses do not show all reads, but merely those that fall within genes (7). Hence the bins of the heatmap do not lie consecutively on the genome (called ‘genomic coordinates’). Instead, each bin covers exactly one gene and the space between genes is not shown in the heatmap. We abstract from the specific type of annotation and allow the use of any type of annotation. We call this coordinate system ‘annotation coordinates’.

To ensure that the render time of our approach is independent of the size of the rendered area, we need to ensure that the number of bins that are displayed is roughly constant. With genomic coordinates, we adjust bin width and height to keep the total number of displayed bins constant. For bins that follow annotation coordinates, we adjust the number of annotations that are displayed within a bin instead. Since prefix sums are stored using genomic coordinates in our index, we implemented a system to translate from annotation to genomic coordinates. For this, we store each annotation type as a sorted array, where each element keeps the genomic start and end position of one annotation instance. Using the first and last annotation contained in a bin, we translate the bin's annotation coordinates to its genomic coordinates for lookup in the index. Combining multiple annotations this way would also count interactions in the void space between the annotations. We hence make use of our annotation filter (described in the Materials and methods section ‘Filtering by annotation using two additional dimensions per annotation type’) to remove these unwanted interactions.

### Reducing file size and preprocessing time

Storing or computing prefix sums for all possible loci in a data space, as suggested above, is not feasible since there are too many loci: We work with a relatively small (compared to human, for example), $\sim 5*{10}^7$nt long assembly of the organism *T. brucei*; Aligners can output 256 different mapping quality values; Rectangles can grow in width and height up to the size of the largest contig, hence $\sim 5*{10}^6$ different values (for *T. brucei*); Finally, one prefix sum requires 32 bytes to store. Factoring up these numbers, we would require $\sim {10}^{20}$ terabytes of storage for one index. Larger assemblies, such as the human genome, would require even more storage. Further, computing this many values would take an insurmountable amount of processing time and power.

Conveniently, reducing the storage requirements and construction time of prefix sum indices has already been studied (17,18). We modified the sparse prefix sum approach of Shekelyan et al. (17) to our needs. They use one lookup table per dimension to translate the original data space into a sparse space. In this sparse space, all empty slices are removed. For example, if there is no point with a $y$ coordinate of 5, the entire slice $y = 5$ is removed in sparse space. These lookup tables span over the entire data space and store, for each position, the number preceding non-empty slices. Any data space coordinate can now be translated into sparse space by lookup in these tables. In sparse space, points are compressed while preserving their order of occurrence in any dimension. It is sufficient to store the prefix sums for all positions in sparse space, merely, since prefix sums cannot change in interaction-free slices.

With an increasing number of datapoints, the efficiency of this compression diminishes. Hence, Shekelyan et al. divide the data space into an even grid of boxes, where each box has its individual sparse space and lookup tables. They pick the number of boxes to optimize the update complexity of the index. In our case, updates are not needed, allowing us to optimize the number of boxes to minimize index size instead. Index size is data-distribution-dependent and thus different for each dataset. We hence implemented an approach that heuristically predicts index size for any given number of boxes. Predicting index size for various numbers of boxes, we determine the minimal size and therefore the optimal number of boxes. Index size is predicted by picking 1000 boxes at random and determining their sparse space as well as the size of their lookup tables. Both values are determined by a binary search over all datapoints. (The datapoints are previously sorted ascendingly for each dimension.) We then extrapolate the average storage space of these boxes to the full index.

To further reduce the required storage size, we divide the $x$ and $y$ positions of all interactions by a constant dividend $d$. This reduces the size of the abovementioned lookup tables. Further, interactions close to the 45$^\circ$ diagonal are often dense enough to make their divided positions concur, reducing the number of non-empty slices and therefore the size of the sparse space. The disadvantage of this approach is that the minimum bin size becomes limited to $d$. We call $d$ the base resolution of the index.

Finally, we also reduce storage size by storing each contig-pair as an individual matrix rather than concatenating them. This reduces index size since non-empty slices from dense contig-pairs (e.g. *cis-*contigs) do not affect sparse contig pairs when stored separately.

### Iterative correction and eigenvector decomposition for partial matrices

State-of-the-art iterative correction (IC), as implemented in e.g. cooler (21), is designed to run on a complete and symmetric Hi-C matrix. However, our index-based approach gains its runtime advantage from merely processing the part of the matrix that is currently visible. Hence, we need to adapt IC to be used on partial matrices. This adaptation is composed of two separate tasks: First, IC needs to work on asymmetric matrices; second, IC needs to approximate the effect of the missing bins.

For dealing with asymmetric matrices, we modified the IC approach to use two sets of biases, one for each dimension. This is necessary since even if the full matrix is symmetric, the currently visible region of that matrix might not be symmetric without the larger context. In Supplementary Note S4, we show that our modification has no effect on symmetric matrices. This proves that the modification is valid.

Second, we sample a subset of the missing bins. A large enough number of samples is expected to even out to the average background of the heatmap. Samples must preserve the rectangular shape of the heatmap and cannot leave empty bins for ICing to work. We hence place samples as evenly spaced concentric rings around the visible area. Here, each ring is one sample that decomposes into several bins to match the columns and rows of the heatmap and previous samples. The bin size of samples is even to the size of bins in the visible area. Sample bins that are outside of the genome are omitted. This way, each sample adds up to two rows and two columns to the heatmap. Supplementary Note S4 gives an example. In the results section, we analyze the number of samples necessary to minimize the noise this sampling-based approach introduces.

### Associated slices normalization for partial matrices

RNA-DNA interactome data can be normalized using the approach of Li et al. (7). This normalization consists of two main steps: First, all interactions with an RNA partner that does not originate from within a chromatin-associated gene body are filtered out. Second, all bins are normalized by the sum of *trans* interactions of chromatin-associated gene bodies for the respective RNA locus.

We now introduce how to compute this normalization without access to the full matrix. First, we determine chromatin-associated gene bodies. To avoid scanning the whole genome, we take evenly-spaced samples of gene bodies (or any type of genomic annotation). In the results section, we analyze which number of samples is necessary to minimize differences between our sampling-based and the global approach. We compute two properties for each sampled gene body: Its average RNA reads per kbp and its maximal DNA reads in a 1 kbp-binned genome. Average RNA reads per kbp are determined by querying the total amount of RNA reads for the gene body (this requires one query per contig, using a bin that spans the gene body on one dimension and the whole contig on the other dimension) and dividing by genome size. Computing the maximal number of DNA reads in a 1 kbp binned genome is not as trivial: Querying every 1 kbp region one by one is not efficient enough. Hence, we use a recursive divide-and-conquer approach: First, we query each complete contig as one ‘region’. We store the queried regions in a max heap, recursively picking out the region with the highest count, splitting it in half, and re-querying it. Once the largest region is of size 1 kbp, we have reached the correct result. Following Li et al.(7) we then proceed to designate chromatin-associated gene bodies by applying thresholds to the average RNA reads and maximal DNA reads.

Next, we compute the RNA coverage of *trans* interactions in chromatin-associated gene bodies for the visible region of the heatmap. For this, we require one query per gene body and visible slice. Finally, we normalize the shown bins by this coverage. Supplementary Note S5 gives an example of this sampling approach. In the results section, we analyze the number of samples necessary to minimize the noise this sampling-based approach introduces.

### Binomial test normalization for partial matrices

RNA-DNA interactome data can be normalized using the approach of Bonetti et al. (6). There, they apply a Binomial test to ascertain whether a bin is statistically significant or not. Following Bonetti et al. (6), we use a Binomial test and correct p-values using the Benjamini/Hochberg method. To approximate applying this normalization to the full matrix, we use a sampling-based approach. We place samples as additional rows above and below the heatmap, where bins in the samples preserve the columns of the heatmap. Supplementary Note S6 gives an example. In the results section, we analyze the number of samples necessary to minimize the noise this sampling-based approach introduces.

### Distance dependent decay normalization for partial matrices

To be able to normalize our matrices by distance dependent decay (DDD), without computing the full matrix, we apply the following scheme: While computing the locations of all bins, we record their distance to the diagonal of the heatmap. Here, the distance can be positive or negative to differentiate the regions right and left of the diagonal (this becomes relevant in the case of asymmetric data). For each recorded distance from the diagonal on each contig-pair, we then sample evenly spaced bins. These sampled bins are of the same size as the displayed bins; They are placed at the recorded distance from the diagonal and spaced evenly in a parallel fashion to the diagonal. We then query the number of interactions in the sampled bins. To compute the DDD for each distance from the diagonal on each contig-pair, we exclude the $x$th top and bottom percentile of sampled bins and average the number of interactions from the remainder. Excluding the top and bottom percentiles of sampled bins is necessary to remove the impact of loops and under-sampled regions, respectively. We compute the average number of interactions instead of the median to deal with sparse data, where most values are zero or one. Such a situation would yield a median that jumps between zero and one, and so, in turn, a noisy picture. Supplementary Note S7 gives an example of this sampling.

### Correcting for ploidy

Typically, interactome data are mapped on a consensus haploid assembly and the analysis assumes that each chromosome is represented an equal number of times. However, organisms can display aneuploidy, where the physical chromosome number is not a multiple of the haploid set. In such cases, the chromosome number of the *in silico* genome assembly would not correspond to the number of physical chromosomes. As a result, some interactions may be over- or underestimated. The same misestimation can arise from chromosomes in the same chromosomal set that contain both homozygous regions, collapsed in the assembly, and heterozygous regions that are assembled as separate contigs. From now on, we will refer to contigs that *in silico* do not match their physical copy number as ‘misrepresented’ and we will call such physical copy number ‘*n*-ploidy’.

To correct for aneuploidy, we implement a strategy that distributes interactions among misrepresented contig-pairs. In detail, we make *n*-ploid contigs appear as *n* ‘instances’ in the heatmap. Then, interactions in misrepresented contig-pairs are divided evenly among the instances of the contigs (Figure 2E). For example, an interaction between a diploid and a triploid contig would count with 1/6 towards each of the 6 (2*3) instances of the corrected contig-pair. An interaction between the same triploid contig and a haploid contig would contribute 1/3 to each of the 3 instances of this contig-pair.

For intra-contig interactions, we assume that all interactions occur intra-instance-wise. Hence, an intra-contig interaction of the triploid contig (with instances A, B, and C) would count 1/3 towards the instance pair A-A, 1/3 towards B-B, and 1/3 towards C-C. The interaction would not count towards any of the other instance-pairs (A-B, B-A, A-C, …). While this assumption does not hold true in nature, it is closer to actual reality than splitting interactions up evenly among all instance pairs.

Finally, we consider the case where chromosomes have varying zygosities. An example is *T. brucei:* Its genome is organized in diploid chromosomes, with homozygous core regions and heterozygous subtelomeric regions (22,23). In the genome assembly, such a chromosome (e.g. chromosome 1) is composed of five contigs: two contigs for the 3′ subtelomere (3′A_chr1_ and 3′B_chr1_), one contig for the core (core_chr1_), and two contigs for the 5′ subtelomere (5′A_chr1_ and 5′B_chr1_). In this case, ploidy correction works by defining the following two groups: 3′A_chr1_-coreA_chr1_-5′A_chr1_, 3′B_chr1_-coreB_chr1_-5′B_chr1_, where coreA_chr1_ and coreB_chr1_ are two instances of the core_chr1_ contig. As with intra-contig interactions, we assume intra-group interactions to take prevalence. In detail, for every contig pair where at least one contig has multiple instances, interactions are split among the pair's instances that are within a group, if at least one pair-instance is within a group. Otherwise, interactions are split among all instances. Hence, interactions occurring between 5′A_chr1_ and core_chr1_ would be assigned to the 5′A_chr1_-coreA_chr1_ instance and not to 5′A_chr1_-coreB_chr1_. Furthermore, interactions between core_chr1_ and 5′A_chr2_, a heterozygous contig of chromosome 2, would be evenly distributed between coreA_chr1_-5′A_chr2_ and coreB_chr1_-5′A_chr2_. Supplementary Note S8 gives an example.

### Virtual 4C

Virtual 4C is an analysis technique where a range on the x- or y-axis of the heatmap is chosen as the viewpoint. The values of bins falling in the range of this viewpoint are then displayed as a one-dimensional graph on the opposite axis. We implement on-the-fly virtual 4C computation. Notably, using prefix sums enables us to place bins such that they span over the complete viewpoint, even for multi-bin-sized viewpoints, making virtual 4C analysis particularly efficient.

### Enhancing the reusability of Smoothers implementation

To enhance the reusability of Smoother, we split our implementation into three different projects: libSps, libSmoother, and Smoother. libSps is a datatype agnostic sparse prefix sum library that is available as a standalone C++17 library, compatible with Windows, Linux, and macOS. It implements multidimensional range count queries on a given set of datapoints or hyperrectangles. Our library can either load the entire index into main memory on startup or use stxxl's (24) cached vectors to load and unload components as required. Keeping the entire index in main memory allows for better runtimes but cannot be done if main memory is smaller than the index. Further, libSps relies on Pybind11, so that it can be imported as a Python 3 module.

Secondly, libSmoother is a C++17 library (compatible with Windows, Linux, and macOS) that takes care of all tasks related to interactome data, such as normalizing, coloring the heatmap, and computing the locations of bins. It makes use of libSps for the computationally expensive task of counting and filtering interactions. To avoid computing values multiple times, libSmoother is implemented as a computational graph. Using this graph, each step is aware of which parameters are relevant to it. Upon a parameter change, only steps that depend on the changed parameter are recomputed. libSmoother also relies on Pybind11 to be importable as a Python 3 module.

Finally, Smoother, our visualization tool, is implemented in Python 3. It makes use of libSmoother as a backend. Further, it uses Bokeh to offer a web browser-based interface, running under Google Chrome, Safari, and Firefox. By being browser-based, Smoother can be deployed both as a webpage or standalone tool.

## Results

### Smoother processes interactome data on-the-fly using a prefix sum index

**Figure 1.**
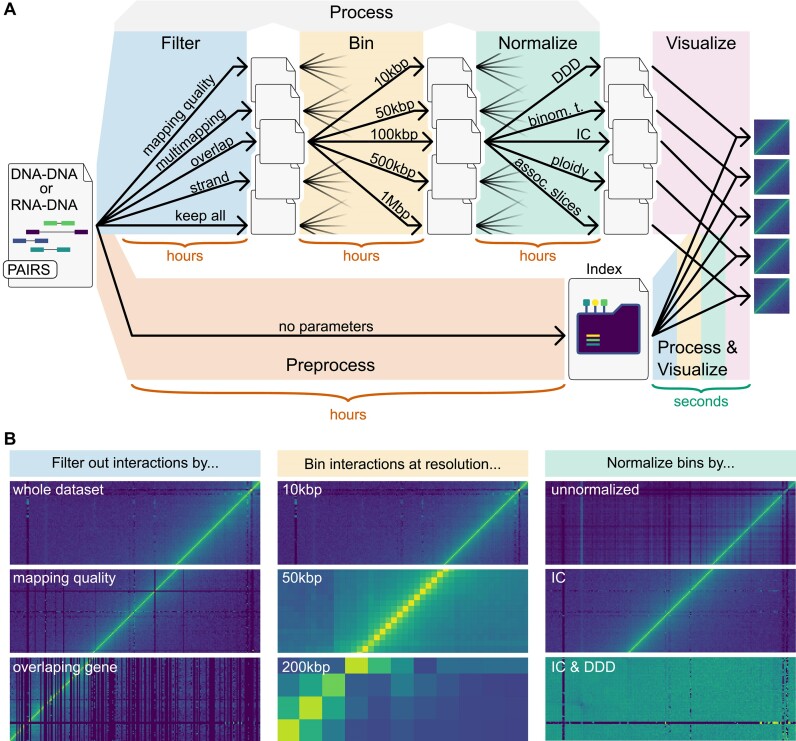
Outline of pipeline-based and index-based data analyses. **(A)** The top path outlines state-of-the-art pipeline-based processing of interactome data. Components of such pipelines are parameterized (black arrows) and can take up to several hours to run. The bottom path outlines our proposed index-based analysis. We preprocess all data into an index in a parameter-independent fashion. Using this index, the actual parameterized data processing can then be done within seconds. **(B)** Parameters of every processing step have a significant impact on the resulting heatmaps. The left panel displays various filters: all interactions, interactions with high mapping quality, and interactions that overlap a gene on the x-axis. The central panel shows interactions binned at various resolutions: 10, 50, and 200 kbp. The right panel illustrates data normalized by various approaches: unnormalized, ICed, as well as both ICed and normalized by Distance Dependent Decay (DDD). All panels show Hi-C data (22) from the central region of chromosome 8 of *Trypanosoma brucei*.

To be able to process interactome data fast enough for an interactive workflow, we broke with the pipeline paradigm that state-of-the-art interactome data analyses follow (10–12) (Figure 1A). Instead, our approach precomputes an index in a parameter-independent manner. Using this index, heatmaps with arbitrary filter parameters, bin size, and normalization, are then generated within seconds (Figure 1B).

Our index uses prefix sums to allow for constant time range sum queries (17,18). A range sum query computes the number of data points that lie in a given range. Importantly, with constant time queries, the time it takes to perform the queries does not increase with variables such as larger bin size, the number of interactions stored in the index, or the size of the analyzed genome. Decoupling the processing time from these variables is what allows on-the-fly data analysis.

### A multidimensional prefix sum index enables various filters

A two-dimensional prefix sum index allows querying the number of interactions in bins with arbitrary sizes and positions. However, it is common to filter these interactions by various criteria. Hence, we extended our prefix sum index to allow such filtering on-the-fly, while keeping the index's runtime properties.

**Figure 2.**
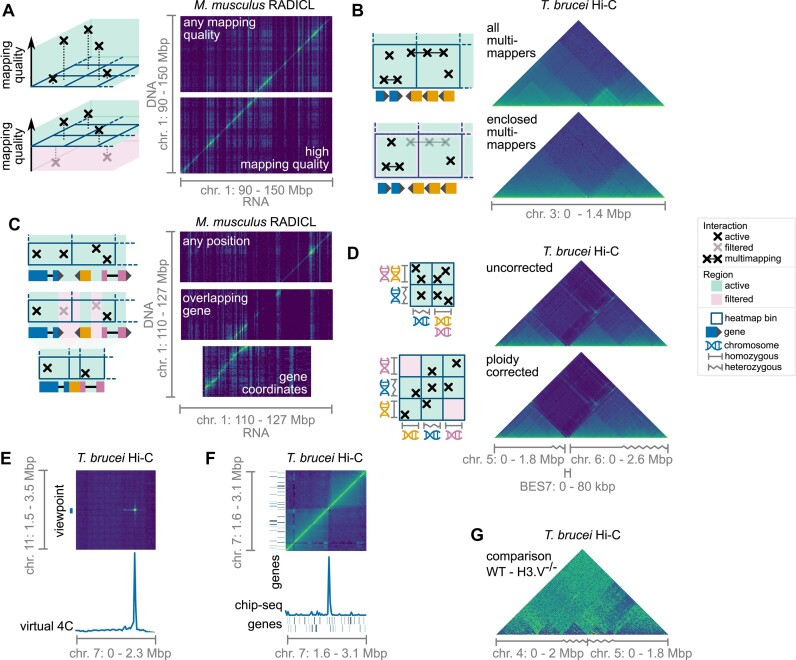
An overview of the on-the-fly data processing that is possible with Smoother. Shown here is *Mus musculus* RADICL-seq data from Bonetti et al. (6) and *T. brucei* Hi-C data from Müller et al. (22). **(A)** Interactions are filtered by mapping quality using a third dimension in the index. **(B)** Multimapping interactions count towards a bin if they are fully enclosed by the bin. This is done dynamically, considering the current bin size. **(C)** Middle panel: Interactions are filtered by whether they overlap an annotation or not. In this example, only interactions that overlap a gene on the y-axis are kept. Bottom panel: Instead of spacing bins out evenly over the genome, each bin is placed on one annotation. In this example, each heatmap column corresponds to a gene. **(D)** Ploidy-corrected heatmaps can be generated. **(E)** A virtual 4C analysis summarizes a slice of the heatmap into a graph. **(F)** ChIP-seq data and genomic annotations are overlaid onto the heatmap. **(G)** Two datasets, e.g. wild type (WT) and H3.V double knockout (H3.V^−/−^) cells, are compared by subtraction.

One of the most common ways to filter interactome data is to remove interactions with low mapping quality. For such quality filtering, we introduced a third dimension to our index, where we place interactions at a position according to their mapping qualities. We then adjust the bounds of the queried region in that dimension to exclude interactions with mapping qualities outside of a desired range (Figure 2A). As before, we use prefix sums, to ensure fast processing times. Interactions with low mapping quality may be multimappers. Multimappers are interactions where one or both of the interaction partners map to multiple loci on the genome. Such multimappers may or may not need to be considered during the analysis. Therefore, we developed a strategy that stores each multimapper as the smallest possible rectangle that encloses all of its mapping loci. Multimappers can then be counted towards a bin if their stored rectangles are fully enclosed by the bin (Figure 2B).

For RNA-DNA interactome data, it is common to filter out interactions that do not align to a gene with their RNA interaction partner (7). We thus introduced a fourth dimension to our index that allows filtering out such interactions. Generalizing from genes, we allow filtering by any type of genomic annotation, on either or both axes (Figure 2C). With all non-gene interactions filtered out, RNA-DNA interactome data is then displayed such that, on one axis, each bin corresponds to one gene (called annotation coordinates), while bins on the other axis are consecutive and cover the whole genome (called genomic coordinates) (Figure 2C). Combining this coordinate system with the annotation overlap filter, Smoother can display heatmaps in the GRID-seq (7) fashion.

Some cell lines and some organisms display aneuploidy or have genomic regions that are highly heterozygous. Both cases result in a situation where the ratio of the genome assembly's contigs to the corresponding physical chromosomes varies. We designed a ploidy correction scheme that splits interactions evenly among such contigs (Figure 2D).

For further downstream analyses, we extended Smoother with several features: First, virtual 4C graphs (Figure 2E) can be computed. Virtual 4C refers to an approach where a slice of the heatmap is displayed as a one-dimensional graph. Typically, it is used to highlight interactions from a specific genomic locus, called the ‘viewpoint’. Next, one-dimensional sequencing data as well as annotations can be overlaid on the heatmap (Figure 2F). Furthermore, two datasets can be compared by subtraction or division (Figure 2G). Finally, Smoother can export publication-quality vector graphics (as SVG files), raster graphics (as PNG files), and raw data (as TSV files).

### On-the-fly normalization of interactome data

To normalize interactome data, we implemented several approaches. For Hi-C data, these include IC (12) and Distance Dependent Decay (DDD) normalization. For RNA-DNA interactome data, we implemented normalization by Associated Slices (7), and Binomial test (6).

**Figure 3.**
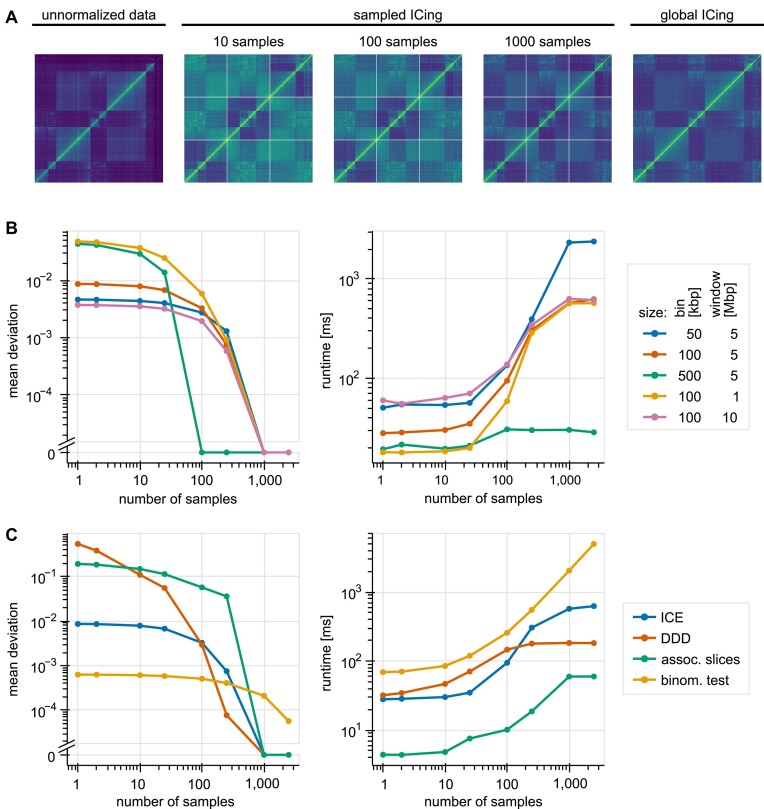
Analyzing sampling-based normalization. **(A)***T. brucei* Hi-C data from Müller et al.(22). The left panel shows unnormalized data, while the right panel shows data that is IC normalized over the entire genome. For the central 3 panels, the genome is split into 5 Mbp-sized windows, which are ICed separately. In addition to the bins within a window, bins of 10, 100, and 1000 sample loci are also considered for ICing. Shown are only the first 3 windows of the full heatmap. **(B)** Left: quantification of A). Mean deviation of heatmap bins is plotted as a function of the number of samples. Running experiments for three different bin and window sizes shows that behavior is consistent across conditions. Right: mean time required to compute one window using default parameters and IC normalization as a function of the number of samples. **(C)** IC and DDD normalization are tested on *T. brucei* Hi-C data from Müller et al. (22), while Associates Slices and Binomial test are tested on *M. musculus* RADICL-seq data from Bonetti et al. (6). Left: Mean deviation of heatmap bins is plotted as a function of the number of samples for various normalizations. A bin size of 100 kbp and a window size of 5 Mbp is used for all approaches. Right: time required to compute one heatmap window using default parameters is plotted as a function of the number of samples for various normalizations. A bin size of 100 kbp and a window size of 5 Mbp is used for all approaches.

In pipeline-based approaches, all these normalizations run on full matrices. However, Smoother achieves its performance by keeping a constant number of bins for every computed heatmap. When visualizing larger sections of a genome, the bin number is kept constant by increasing the bin size. Further, when zooming in to look at smaller regions of a genome, a constant number of bins is maintained by omitting bins outside of these regions of interest. This omission of bins prevents the use of normalization techniques that are designed to work on a full matrix. To solve this problem, we extended normalizations with a sampling strategy that considers some bins outside of the visible area during normalization. We argue that normalizations will behave as if they were applied to the full matrix, given a large enough number of samples. To evaluate our approach, we subdivided matrices into evenly sized windows. We then normalized each of those windows separately and concatenated them back together before comparing them to a matrix that was normalized in its entirety. For all normalizations, we find that normalizing with an increasing number of samples approaches normalizing over the whole matrix (Figure 3A and Supplementary Note S9). To quantify these results, we computed the mean deviation of bin values from heatmaps normalized in windows and globally. Next, we plotted the mean deviations as a function of the number of samples (Figure 3B). For all normalizations, we find the mean deviation to decrease with an increasing number of samples (Figure 3C). While IC, Associated Slices, and DDD reach a deviation of zero with $ \sim$1000 samples, Binomial test approaches zero more slowly. While varying the bin and window size may shift deviation up or down, neither variable affects the general trend that is observed (Figure 3B and Supplementary Note S9). To obtain a good balance between accurate normalization (large number of bins sampled outside the visible area) and fast processing speed (small number of bins sampled outside the visible area), we set the following number of samples as default: 300 samples for IC, 1000 samples for Associated Slices, 100 for Binomial test, and 200 for DDD normalization. However, the number of samples can be adjusted in Smoother on-the-fly.

Further, for 3C normalizations, we extended the approaches to deal with asymmetric data. This extension is necessary since subsections of a symmetrical matrix that are not centered on the diagonal are asymmetrical, even with our sampling approach. Additional details on how normalizations are extended and how their sampling is implemented can be found in the Materials and methods section.

### Benchmarking of Smoother reveals processing times in the millisecond range

We benchmarked Smoother, analyzing processing times, index size, and preprocessing times. To obtain an overview of our approach for different datasets, we varied the base resolution of our index, the number of interactions in the dataset, the reference genome, and the set of activated filters.

**Figure 4.**
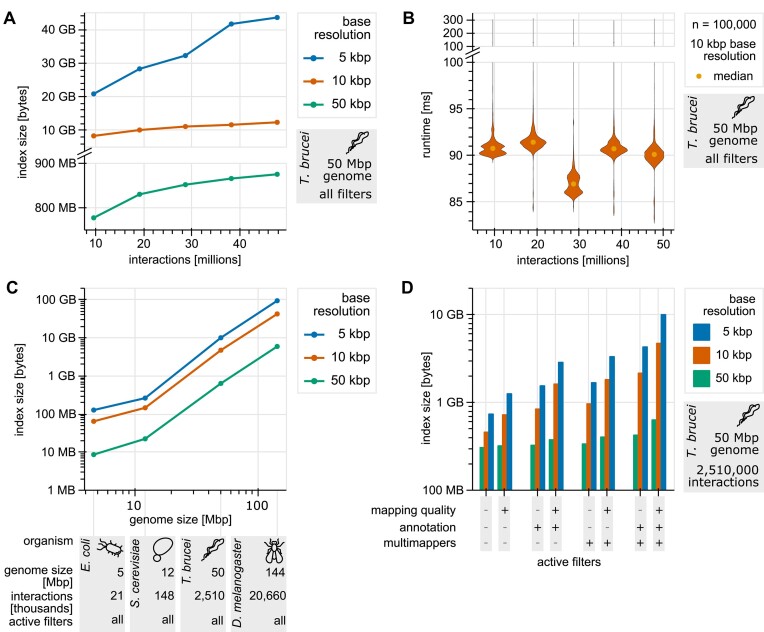
Benchmarking index size and speed. **(A)** Index file size as a function of the number of stored unique interactions. **(B)** Time required to compute one heatmap using default parameters as a function of the number of unique interactions in the index. **(C)** Index file size as a function of the genome size. **(D)** Index file size for different active and inactive filters.

To evaluate index file size, we first merged two wild-type *T. brucei* Hi-C datasets (genome size $\sim$50 Mbp, number of interactions: $\sim$50 million) (22,23). By subsampling from the merged dataset to 20%, 40%, 60%, and 80%, we show that index file size increases at a decreasing rate with the number of interactions in the dataset (Figure 4A). This slowdown continues until every bin contains at least one interaction. At this point, the index is saturated. Adding further interactions would merely increase the values of the prefix sums in the file but not introduce new bins that need to be stored. Hence, once saturation is reached file size does not increase any further. Indices can be built with varying base resolutions. The base resolution of an index is the highest resolution the index can be used to create heatmaps. In theory, base resolution can reach up to a single base pair; however, practically, higher base resolutions increase file size, so limiting the base resolution to for example 5 kbp is reasonable. Additionally, we showed that index preprocessing time is relative to the file size (Supplementary Note S10–Supplementary Figure S14A).

Next, we measured processing times for 1000 heatmaps with Smoother's default parameters. Here, we measured the time required to compute a heatmap. We show that, as expected using prefix sums, processing time stays constant when the number of stored interactions increases across various base resolutions (Figure 4B and Supplementary Note S10–Supplementary Figure S14B).

To measure the effect of varying genome size, we obtained Hi-C data from *Escherichia coli* (25), *Saccharomyces cerevisiae* (26), *T. brucei* (22,23), and *Drosophila melanogaster* (27) and down-sampled the datasets to an even density of 1000 interactions per squared mega-base pair. Such even density is crucial to measure the effect of varying genome size exclusively instead of measuring a combined effect of genome size and number of interactions in the dataset. We then measured index file size for all datasets (Figure 4C) and find that file size grows linearly with genome size, except for very small genomes. This demonstrates the effectiveness of the applied index compression, as an uncompressed index would have a file size that scales with the square of the genome size (see the Methods section ‘Reducing file size and preprocessing time’). Again, we confirmed that preprocessing times correlate with file size (Supplementary Note S10–Supplementary Figure S14C) and that processing speed is roughly constant (Supplementary Note S10–Supplementary Figure S14D) for varying genome sizes.

Finally, we measured the effect of our filters on the index file size, using the *T. brucei* dataset. For this purpose, we removed the information relevant to the filters from the dataset before generating the index. For example, for the mapping quality, we placed all interactions at a default value in the mapping quality dimension. Comparing an index with filters to one without filters, we observe a ∼10-fold file size (Figure 4D) and ∼3-fold preprocessing speed (Supplementary Note S10–Supplementary Figure S14E) difference. Filters increase index size, as each interaction is placed at a position representing its value for the corresponding filter in the index. For inactive filters, however, interactions are placed at a default value, hence reducing the size of the area that is covered by the index. Accordingly, we see index size vary significantly; however, processing time increases only minimally (∼1.5-fold; Supplementary Note S10–Supplementary Figure S14F). Supplementary Note S11 gives an overview of the number of dimensions used for each filter.

In summary, Smoother's processing time stays almost constant as the amount of data increases, regardless of whether the increase is in the number of interactions, genome size, number of filters, or base resolution. Instead, the computational overhead of increased data amount now affects the file size of the index. The most significant increase in file size is observed with increasing genome size and base resolution. Even though each filter has a significant impact on the file size, the overall effect is small because the number of filters is small. Further, large numbers of interactions can be tolerated better than large genome sizes. However, we obtained reasonable index file sizes for any datasets with genomes smaller than 150 Mbp thus making Smoother perfectly suited for the analysis of archaea, bacteria, most fungi, nematodes, and many protists.

## Discussion

Here, we developed Smoother, a tool to analyze and visualize nucleic acid interactome data on-the-fly. To our knowledge, Smoother is the first tool that offers ultrafast, on-the-fly analysis of interactome data. This analysis includes, among others, filtering interactions by their mapping quality, and by whether they overlap annotations. Further, Smoother rescues multimapping interactions, corrects for ploidy, normalizes with various methods, compares different datasets, and offers downstream analyses such as virtual 4C.

Smoother is based on prefix sums. We chose this approach as it minimizes the runtime required to query interaction counts. In contrast, storing interactions as a count matrix, as is done for pipeline-based approaches (20,21,28), requires storing bins for each permutation of filter values. While querying bins, relevant permutations then have to be summed up, slowing down runtime based on the chosen filter thresholds. To maintain this theoretical runtime advantage of prefix sums in real-world applications, index compression must be chosen accordingly. For example, Shekelyan et al.’s (17) approach is suited for this, since they maintain linear query complexity. In contrast, sparse coordinate lists, the approach that is commonly used to store count matrices (21), increase the runtime complexity. While either compression could be applied to both prefix sum and count matrices, we chose Shekelyan et al.’s approach for its better runtime behavior despite its lower compression rate. In addition to the lower compression rate, index size is increased by prefix sums requiring more bins to be stored than count matrices (Supplementary Note S12). However, the improved runtime behavior by prefix sums and the chosen compression, combined, allows on-the-fly data analysis.

Further, prefix sums remove the need to coarsen heatmaps to various resolutions as is done with count matrices (21). Prefix sums even supersede the abilities of coarsening and allow computing bins at any resolution and position, whereas coarsening limits the chosen resolutions and bin positions.

Since our approach is optimized for runtime complexity (speed), the file size of our index becomes a limiting factor. For species with large genomes, such as the human genome, even indices with a low base resolution of 50 kbp are larger than 100 Gb. File size can be reduced significantly by deactivating filters; however, this will also lead to Smoother losing some of its on-the-fly analysis capabilities. Instead of deactivating filters, future research could try to optimize the number of dimensions used by the individual filters. There are several reasons analysis tools based on count matrices are better at handling large genomes than our prefix sums based approach. Primarily, count matrices’ can be compressed more strongly. In addition, fewer interactions are stored in count matrices, as filtering is done beforehand, removing some interactions from the file. Finally, less data is stored for each interaction. For example, mapping quality, annotation overlap, and multimapping loci are not tracked.

In summary, Smoother is ideally suited to analyze interactome data of many invertebrate genomes, such as those from many unicellular parasites like *Plasmodium falciparum*, *Toxoplasma gondii*, *Trypanosoma brucei*, many yeast species, and bacteria, where optimal analysis parameters are uncertain (25,26,29,30). Due to the integrated ploidy correction, Smoother is further excellently suited to analyze aneuploid organisms such as *Trypanosoma cruzi* and *Leishmania spp*. The ability to quickly compare interaction maps containing all reads with those only containing uniquely mapping reads, to instantaneously visualize the effect of different normalization methods and the ability to rescue a large proportion of non-uniquely mapping reads opens new possibilities to evaluate interactome maps. We believe that these features are especially important for genomes containing many repeats that have complicated analyses in the past. While ideally suited for genomes < 150 Mbp, Smoother can also be used for larger genomes if subsections of the genome are analyzed as with methods such as capture Hi-C (31), or Micro Capture-C (3), or if datasets contain fewer than 20 million interactions such as with some RNA-DNA interactome capture methods. Ongoing research aimed at improving the compression of prefix sums will open the possibility of using Smoother for genome-wide analyses of larger genomes.

## Supplementary Material

gkae008_Supplemental_File

## Data Availability

No data was created for this project. The used interactome datasets are publicly available under the Sequence Read Archive accession codes: SRR5820092 (*D. melanogaster*), SRR6354577 (*E. coli*), SRR14469367 (*S. cerevisiae*), SRR7721317, SRR7721318 and SRR7721307 (*T. brucei*), as well as SRR9201799 and SRR9201800 (*M. musculus*). The used genome sequences and their annotations are publicly available on NCBI under the RefSeq assembly Ids: GCF_000001215.4 (*D. melanogaster*), GCF_000005845.2 (*E. coli*), GCF_000146045.2 (*S. cerevisiae*), and GCF_000001635.27 (*M. musculus*). The *T. brucei* genome sequence and its annotations are publicly available on TryTrypDB under www.tritrypdb.org/common/downloads/Current_Release/TbruceiLister427_2018/. Smoother is available as a pip package using ‘pip install biosmoother’. Source code is available on GitHub at www.github.com/Siegel-Lab/BioSmoother, www.github.com/Siegel-Lab/libBioSmoother, and www.github.com/Siegel-Lab/libSps, for Smoother, libSmoother, and libSps, respectively. Further, all code has been deposited on Zenodo: Smoother: https://doi.org/10.5281/zenodo.8402173, libSmoother: https://doi.org/10.5281/zenodo.8402040, and libSps: https://doi.org/10.5281/zenodo.8386312. All code is published using the MIT license. Benchmarking results can be reproduced using the scripts in the benchmarking directory of the libSmoother repository. Throughout the manuscript, Smoother version 1.0.0, libSmoother version 1.0.0, and libSps version 1.0.0 were used.
