# Supplementary material for: Smoother: on-the-fly processing of interactome data using prefix sums
Source: Nucleic Acids Res. 2024 Jan 28;52(5):e23. doi: 10.1093/nar/gkae008 (PMC10954447; doi:10.1093/nar/gkae008)
Supplement: gkae008_Supplemental_File [file gkae008_supplemental_file.pdf]

Smoother: On-the-fly processing of interactome data using prefix sums.

Markus R. Schmidt[1,2,*], Anna Barcons-Simon[1,2], Claudia Rabuffo[1,2], and T. Nicolai Siegel[1,2,*]

[1]Division of Experimental Parasitology, Faculty of Veterinary Medicine, Ludwig-Maximilians-Universität München, Munich, Germany

[2]Biomedical Center, Division of Physiological Chemistry, Faculty of Medicine, Ludwig-Maximilians-Universität München, Munich, Germany

*Correspondence: markus.schmidt@lmu.de, n.siegel@lmu.de

Supplementary Notes

Supplementary Note 1 – d-dimensional prefix sums

Below we give an example of the 2-dimensional case. Interactions are shown as orange and green crosses, prefix sums as numbers. For performing 2-dimensional counting operations, 4 prefix sums need to be considered: A 2-dimensional bin is a rectangle (the bin is drawn as a dashed line). The prefix sum at each corner of the rectangle is looked up. By adding up the prefix sums of the top-right and bottom-left corners and subtracting the prefix sums of the top-left and bottom-right corners, the number of interactions within the bin is obtained. This pattern of additions and subtractions is necessary to count all interactions below the top-right corner of the bin, but ignore all interactions to its left (top-left corner) and bottom (bottom-right corner). Since the region to the bins left and bottom overlap, the bottom-left corner prefix sum is used to re-add the interactions that were subtracted twice.

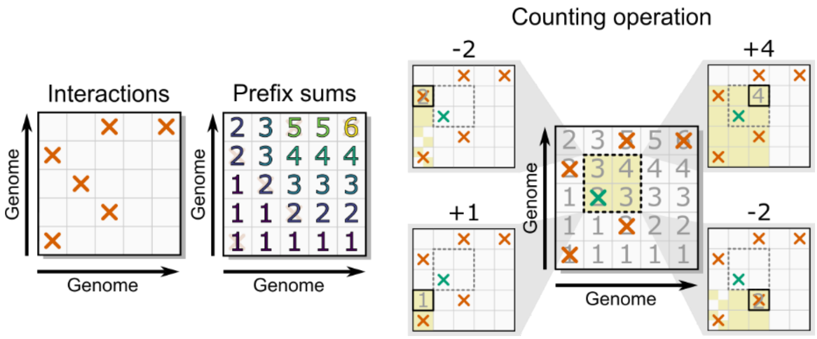

Supplementary Figure 1. The prefix sums (center) for a set of interactions (left). The 4 lookups that are required to query a rectangle (right).

This pattern also holds for the d-dimensional case. We now give a generalized formal definition. Let I be a set of d-dimensional interactions. The prefix sum matrix P contains, for each position, the number of interactions that are located at or before that position in each dimension. Formally, we define:

$$P(p_1, \ldots, p_d) = \sum_{(x_1,\ldots,x_d)\in I} 1[x_1 \leq p_1, \ldots, x_d \leq p_d],$$

Where $P\left(p_1, ..., p_d\right)$ is the prefix sum in P at position $\left(p_1, ..., p_d\right)$ and $\left(x_1, ..., x_d\right)$ is the position of an interaction in I.

The number of points in a hyperrectangle with the lower corner $\left(l_1, ..., l_d\right)$ and upper corner $\left(u_1, ..., u_d\right)$ can be counted by summation/subtraction of the prefix sums for the hyperrectangle's corners. Essentially, the prefix sum of the upper corner $\left(u_1, ..., u_d\right)$ holds the number of points within and before the hyperrectangle. The prefix sums of the other corners (e.g. $\left(l_1, u_2, ..., u_d\right)$, $\left(u_1, l_2, ..., u_d\right)$, ...) hold some fraction of the points before the hyperrectangle. However, these fractions of points also overlap in various ways. To cancel out these overlaps, we combine the prefix sums of all corners where prefix sums from corners with an even distance to the upper corner are summed up, while those with an uneven distance are subtracted. Here, distance between two corners denotes the minimal number of edges that separates them. Formally, we count the number of points between $\left(l_1, ..., l_d\right)$ and $\left(u_1, ..., u_d\right)$, by:

$$f((p_1, \ldots, p_d),\ (u_1, \ldots, u_d)) \ = \begin{cases} 1 & if \left(\sum_{i \in [1,d]} 1[p_i = u_i]\right) mod\, 2 = 0 \\ -1 & else \end{cases}$$

$$\sum_{p_1 \in \{l_1, u_1\}} \cdots \sum_{p_d \in \{l_d, u_d\}} P(p_1, \ldots, p_d) \cdot f((p_1, \ldots, p_d),\ (u_1, \ldots, u_d)) \quad .$$

Using prefix sums, the time required to count the number of interactions in any given interval, rectangle, cuboid, or d-hyperrectangle is independent of the hyperrectangle size or the size of the dataset. It always requires 2^d lookups. E.g., for intervals, 2 lookups are required (start and end position of the interval), while our 2-dimensional contact data requires 4 operations per bin.

Supplementary Note 2 – Alternative data structures to prefix sums

Prefix sums are not the only data structure that offers fast region count query speeds. Other options are R-trees or range trees. The main reason to use prefix sums is that they offer the best query times out of the three options. Additionally, prefix sums lend themselves nicely to our task, while the other listed data structures have particular caveats.

R-trees recursively group nearby points into bounding boxes. Queries are performed by traversing down the tree into bounding boxes that match the search while ignoring the boxes that do not. For region count queries, one could annotate each bounding box with a counter that stores the total number of points inside; hence a count query that fully encloses a bounding box would not need to traverse into that box.

Range trees, such as e.g. k-d trees, are binary search trees that partition data points using a different dimension of these points at each layer of the tree. Similarly to R-trees, nodes can be annotated with counters to store the total number of datapoints below them, removing the need to traverse past nodes that are fully enclosed by the query range.

However, with both R-trees and k-d trees, the surface of the queried region will very likely not match any of the stored bounding boxes or nodes, even on the lower layers of the tree. Hence, for all bounding boxes or nodes that overlap the surface of the queried region, it will be necessary to descend to the

lowest layers of the tree and count the stored points individually. When computing a heatmap that covers the entirety of the genome, these surface-overlapping bounding boxes are expected to significantly slow down these approaches.

Even in an ideal case, where the bounding box or node surfaces are aligned to the queried area, a lookup in these tree based datastructures requires descending down the tree, making prefix sums the superior datastructure for our purpose.

Supplementary Note 3 – storing hyperrectangles using prefix sums

Below, we give an example for 2-dimensional multimapping interactions. Multimappers are shown as orange and green rectangles. Each rectangle is the smallest rectangle that surrounds all mapping loci of the multimapping interaction. The 4 outer panels show the counting operations performed for the four corners of a bin (dashed rectangle). For each corner of the bin, we count the number of rectangles that have the equivalent corner to the bottom left of the bin's corner. Here, a black cross marks the corner of the bin, while the equivalent corners of all rectangles are indicated with colored crosses.

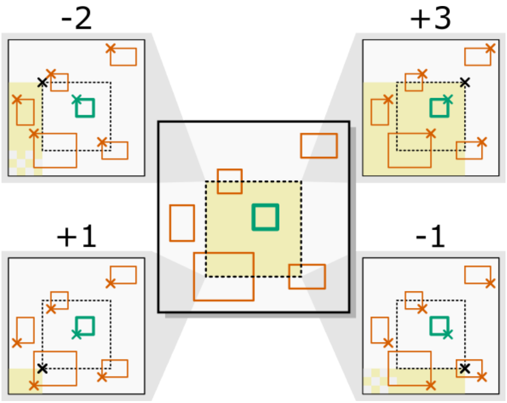

Supplementary Figure 2. A diagrammatic representation of querying data rectangles in a prefix sum index.

This counts merely rectangles that are fully enclosed by the bin if no rectangle fully encloses the bin. To filter out such enclosing rectangles, we filter out all rectangles that are wider or higher than the bin. To do this we introduce two new dimensions. Below we give a one-dimensional example, where intervals that are fully enclosed by a bin are counted. For this, we introduce a second dimension, where intervals are stored at a position according to their width. While querying a bin, we then adjust the top face of the bin to exclude too large intervals.

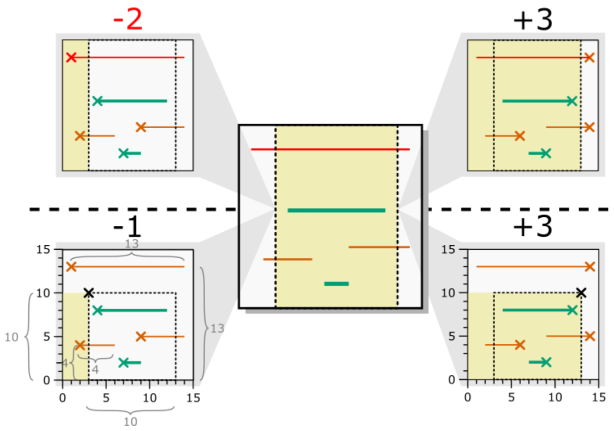

Supplementary Figure 3. A filter dimension is used to exclude data intervals that are larger than the query interval.

Since the bottom edge of the query rectangle is always at zero for this filter dimension, the prefix sums of these edges' points must be zero and need not be queried.

For counting enclosed data rectangles, we pick the same corner that is used for the query rectangle. However, it is also possible to count rectangles that overlap the queried bin by picking the opposite corner and skipping filtering by rectangle width and height.

This pattern also holds for the d-dimensional case. We now give a generalized formal definition. Let I be a set of e-dimensional hyperrectangles, in d-dimensional space, defined by their lower $\left(a_1, ..., a_d\right)$ and upper corners $\left(b_1, ..., b_d\right)$. Additionally, we require that the dimensionality of data hyperrectangles is lower than the dimensionality of the dataspace $e \leq d$ (meaning data hyperrectangles are allowed to be placed in higher dimensional space, being flat in the dimensions they do not share with the dataspace). For non-hyperrectangle dimensions, the upper and lower corner coordinates must be equal $\left(a_{e+1}, ..., a_d\right) = \left(b_{e+1}, ..., b_d\right)$.

Further, we add e more filter dimensions to our data space (one for each of the data hyperrectangle dimensions). These dimensions will be used to filter out data hyperrectangles larger than the query hyperrectangle (see the second figure). For these filter dimensions data hyperrectangles are flat and placed at a position matching their width in the corresponding regular dimension $\left(a_{d+1}, ..., a_{d+e}\right) = \left(b_{d+1}, ..., b_{d+e}\right) = \left(a_1 - b_1, ..., a_e - b_e\right)$.

We compute the prefix sums, individually for all 2^e corners of the hyperrectangles. We denote these corners (and so prefix sum sets) by an e-tuple $t_1, ..., t_e$, with $t_i \in \{\top, \perp\}$, for upper and lower corners, respectively. We hence obtain a set of prefix sum indices

$$P^{\top_1,\top_2,...,\top_e}, P^{\perp_1,\top_2,...,\top_e}, P^{\top_1,\perp_2,...,\top_e}, ..., P^{\perp_1,\perp_2,...,\perp_e}.$$

We query the number of enclosed data hyperrectangles in a query hyperrectangle defined by its lower $\left(l_1, ..., l_d\right)$ and upper corner $\left(u_1, ..., u_d\right)$ as follows:

$$f((p_1, \ldots, p_d),\ (u_1, \ldots, u_d)) = \begin{cases} 1 & if \left(\sum_{i \in [1,d]} 1[p_i = u_i]\right) mod\, 2 = 0 \\ -1 & else \end{cases}$$

$$\sum_{t_1, p_1 \in \{(\bot, l_1), (\top, u_1)\}} \cdots \sum_{t_d, p_d \in \{(\bot, l_d), (\top, u_d)\}} P^{t_1, \ldots, t_e}(p_1, \ldots, p_d, u_1 - l_1, \ldots, u_e - l_e) \cdot f((p_1, \ldots, p_d), (u_1, \ldots, u_d))$$

.

Supplementary Note 4 – IC sampling

IC samples are placed as concentric rings around the visible area. Each sample is one ring that consists of multiple bins. Bins in a ring are placed in such a way that the columns and rows of the visible bins and previous samples are conserved. The void space between the samples is chosen so that samples evenly cover the genome. Bins of rings that would be placed past the end of the genome are omitted. In this example, only the first 3 samples are shown.

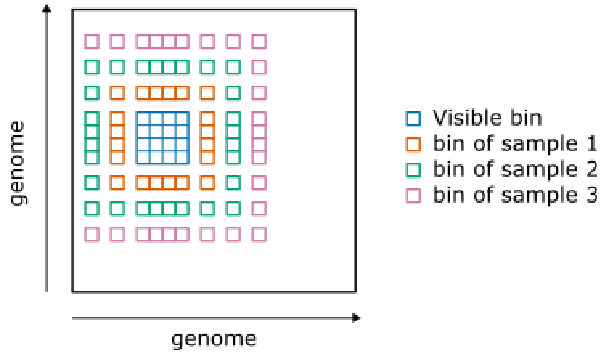

Supplementary Figure 4. Samples for IC normalization are distributed as concentric rings around the visible region of the heatmap.

In addition to sampling, we modified the IC implementation to use two sets of biases, one for the x axis and one for the y axis. To check the effect of this modification, we used a symmetric Hi-C matrix of *T. brucei* data. For 50, 100, and 500 kbp bins, we normalize the whole matrix using our modified approach and the IC implementation ("balance_cooler") in cooler version 0.9.1. Comparing the values of all bins, we then found that both implementations produce the exact same result.

Supplementary Note 5 – Associated slices sampling

Below we give an example of the sampling for the associated slices normalization. First, the average RNA reads per kbp are determined for each sample. In the example, every second gene is used as a sample. Average RNA reads are determined by querying the total RNA reads, which requires one bin per gene and contig (contigs are drawn as light gray rectangles).

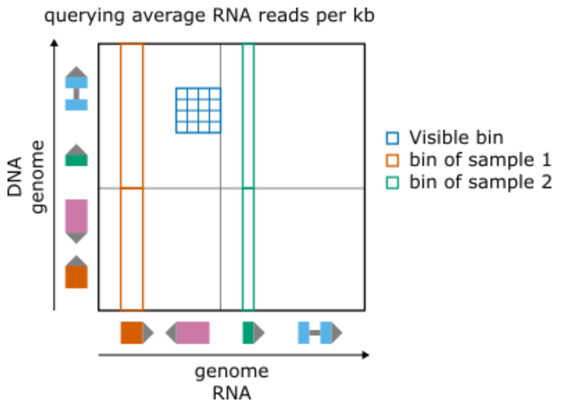

Supplementary Figure 5. Chromatin associated slices are determined by querying their average RNA reads per kb using one bin per sample and contig.

Next, for each sample, the maximal DNA reads per kbp are determined. For this, we use an iterative approach. First, for each sample, we stretch one bin over each contig. Then we repeatedly take the bin with the largest count (counts are annotated in the figure) and split it in half. Once the bin with the largest count reaches the size of 1 kbp, we stop the repetition. Using this approach, we guarantee to find the bin with the largest count but avoid checking every bin one by one.

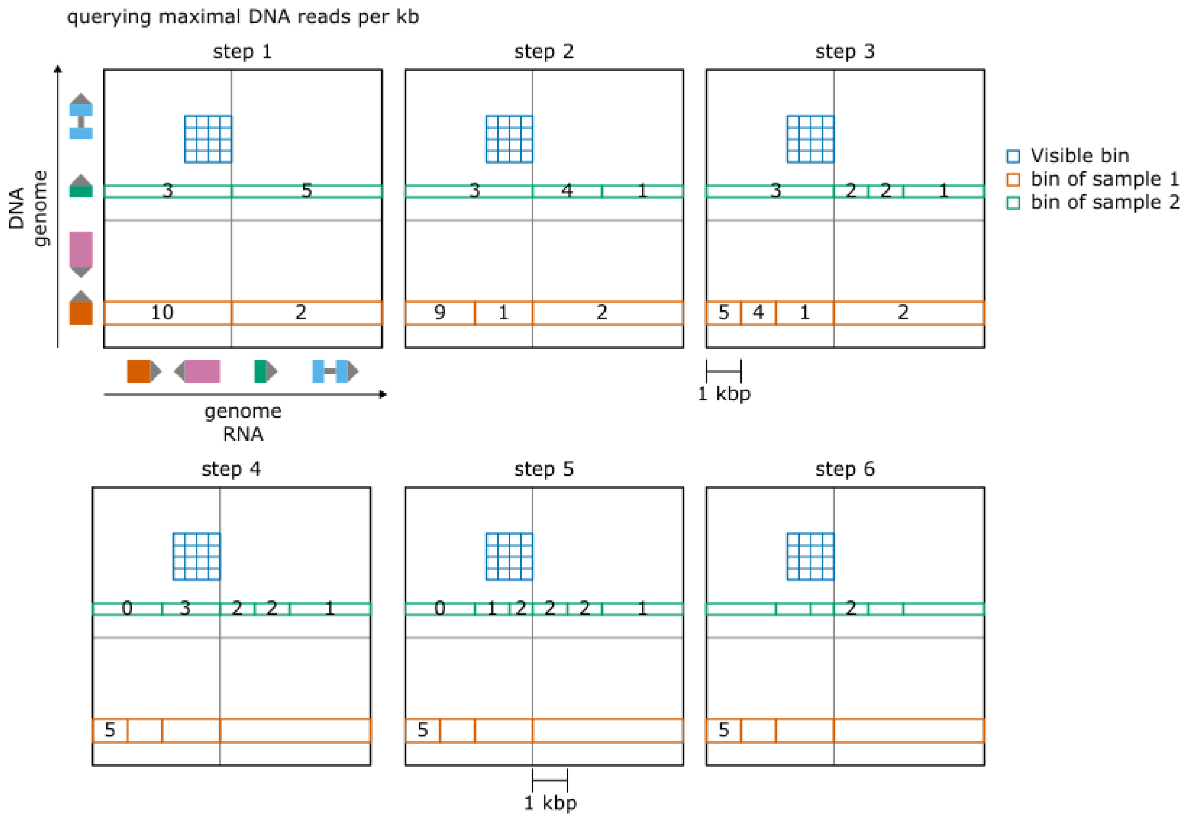

Supplementary Figure 6. Chromatin associated slices are determined by querying their maximal DNA reads per kb using a divide-and-conquer approach.

Finally, we filter our samples as described by Li et al.[1], based on the above two values. In our example, neither sample was filtered out. From the remaining samples, we sum up all *trans* interactions to compute an RNA coverage. We normalize the visible bins by this RNA coverage.

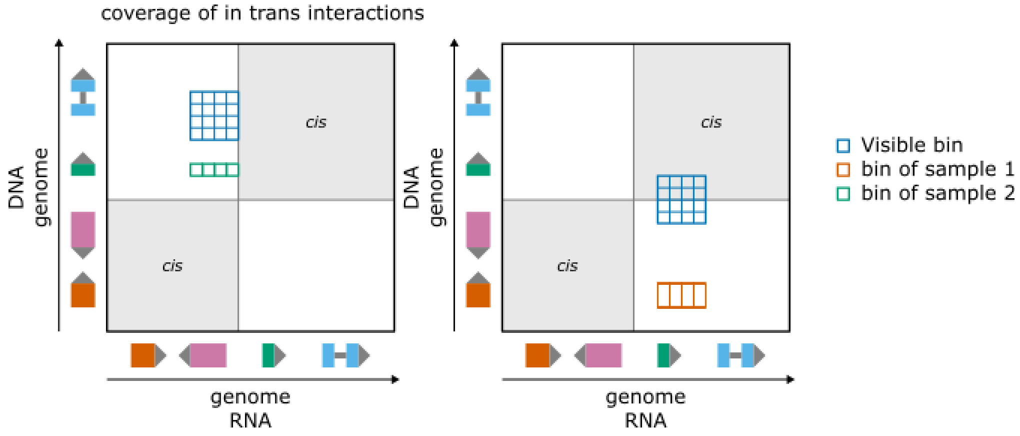

Supplementary Figure 7. Samples for the Associated slices normalization are placed one bin per column of the visible region of the heatmap and chromatin associated slice.

Supplementary Note 6 – Binomial test sampling

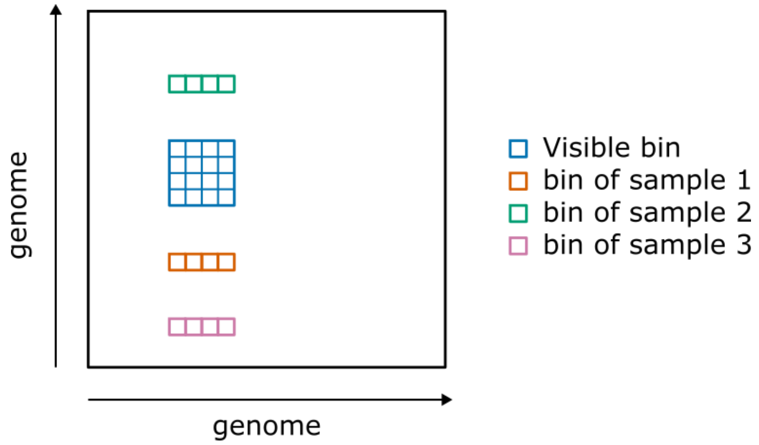

Supplementary Figure 8. Samples for the Binomial test normalization are placed as additional rows of the heatmap.

Supplementary Note 7 – Distance Dependent Decay normalization sampling

Below we give an example for the sampling of our distance dependent decay normalization. In the example, two of the visible bins are highlighted in blue. Those two bins are on the same 45° diagonal. We show how three samples would be spaced out for these two bins. Visible bins on different diagonals would have their own set of samples.

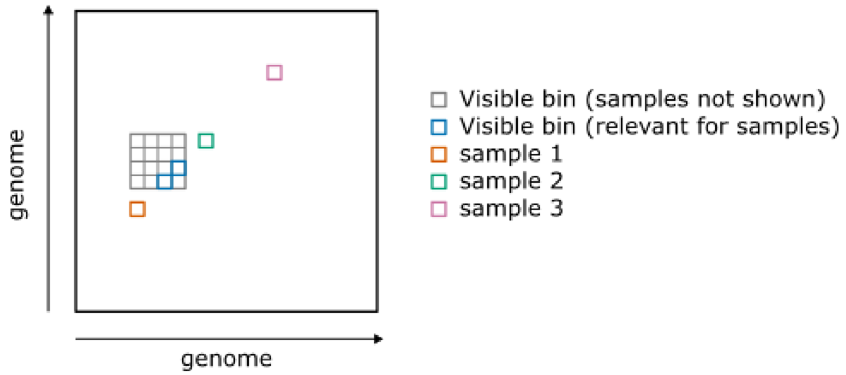

Supplementary Figure 9. Samples for the DDD normalization are placed along the diagonals of the visible bins.

Next, from all samples, we exclude the *x*th bottom and top percentile (left panel). This removes outliers such as loops and undersampled bins. From the remaining bins, we compute the mean value. We compute the mean instead of the median in order to deal with regions with little interaction coverage. In these regions, many bins might have zero or one interaction (right panel). Taking the median in such cases would result in a very noisy heatmap, where some diagonals are divided by a high number and some by a low number.

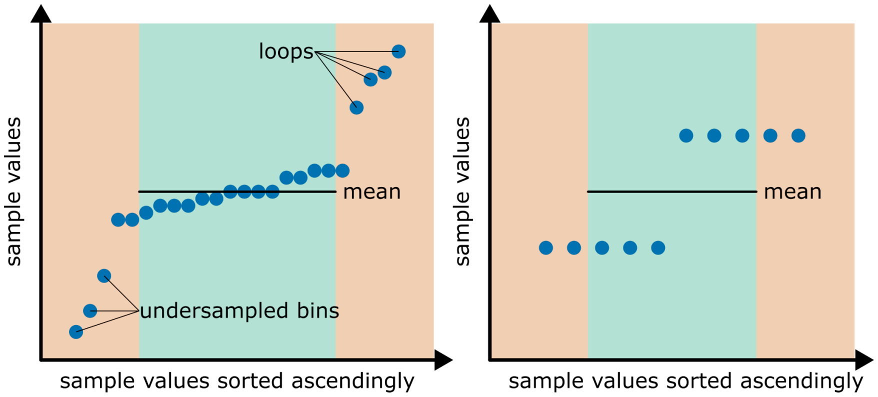

Supplementary Figure 10. Some samples for the DDD normalization excluded using a percentile based approach before computing their median value.

Supplementary Note 8 – Ploidy correction example

Below we draw the two ploidy correction examples from the methods section of the main text. Instance-pairs are drawn as black squares, where the halves are colored according to the contig they belong to. In the uncorrected matrix, each contig has one instance, hence each contig-pair has one instance-pair. In the corrected matrix, *n*-ploid contigs have *n* instances. Interactions are distributed evenly among the valid instance-pairs of each contig-pair. In the figure, the numbers indicate the fraction that the stored interaction count is divided by to correct the matrix.

The validity of instance-pairs is decided as follows:

```
For a given instance-pair, where each instance originates from one contig; and
a set of groups, where each instance belongs to exactly one group:
      if the instances are within the same group:
            Pair is valid
      else:
            if the instances never appear in the same group:
                  Pair is valid
            else:
                  Pair is not valid
```

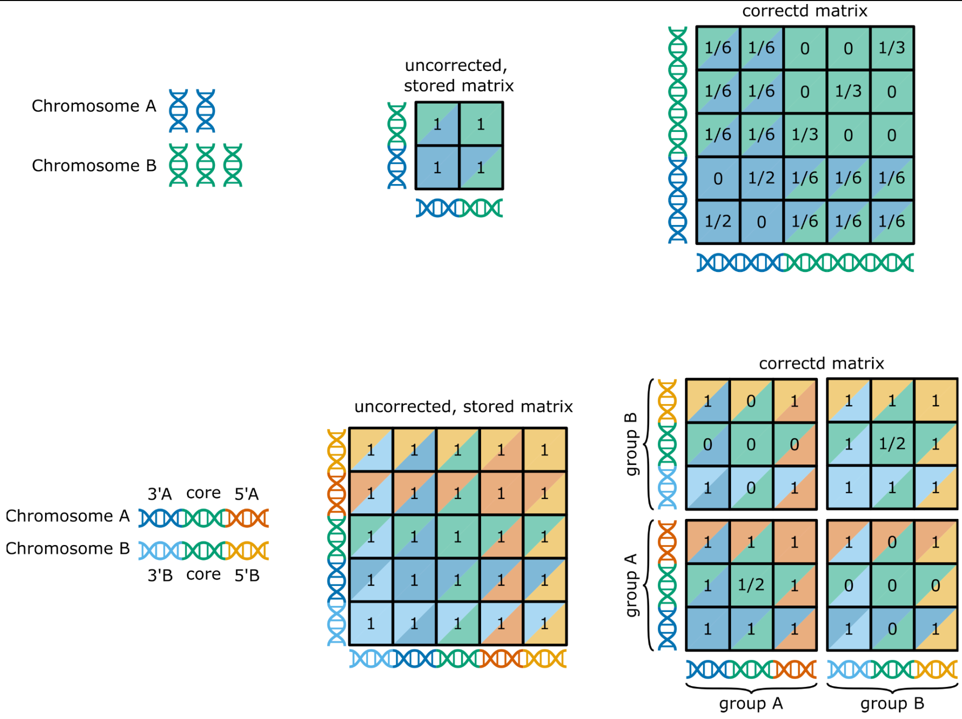

Supplementary Figure 11. A diagrammatic representation of the ploidy correction example from the main text.

Supplementary Note 9 - Extended normalization analysis

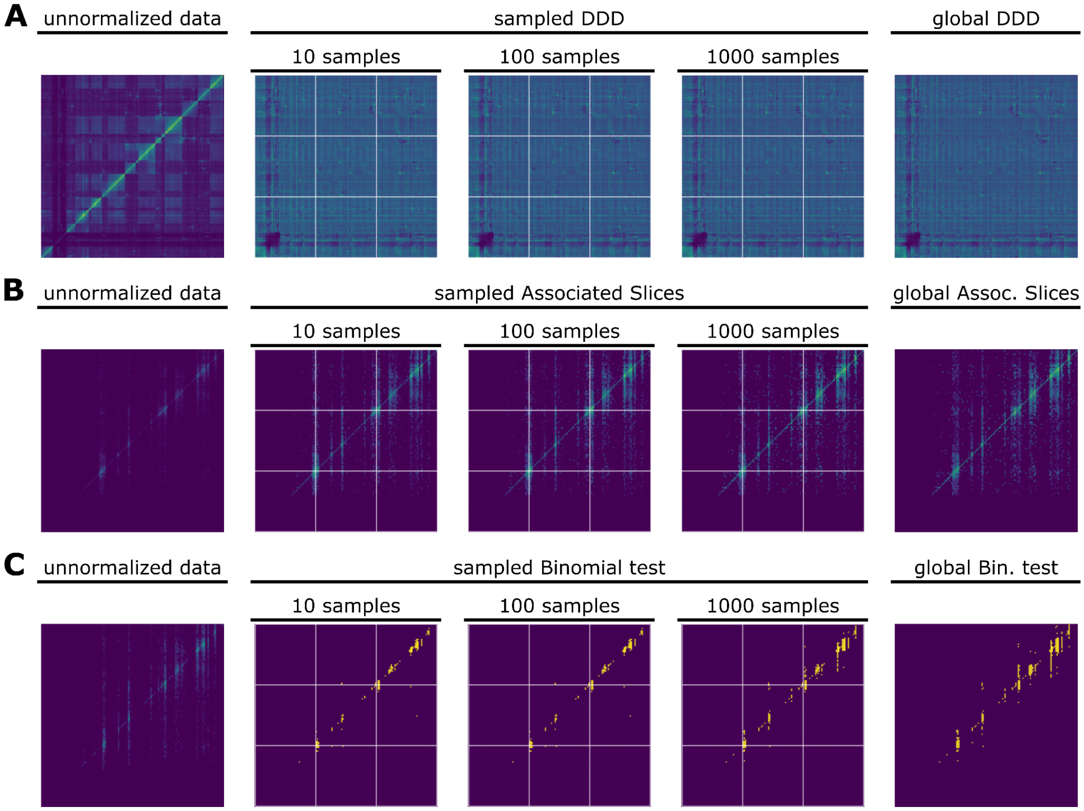

Supplementary Figure 12. Extended version of Figure 3A of the main text. We show *T. brucei* Hi-C data in A) and *M. musculus* data in B) and C). All panels show unnormalized data and data normalized using 10, 100, and 1000 samples as well as the whole heatmap. (**A**) DDD normalization. (**B**) Associated Slices normalization. (**C**) Binomial test normalization.

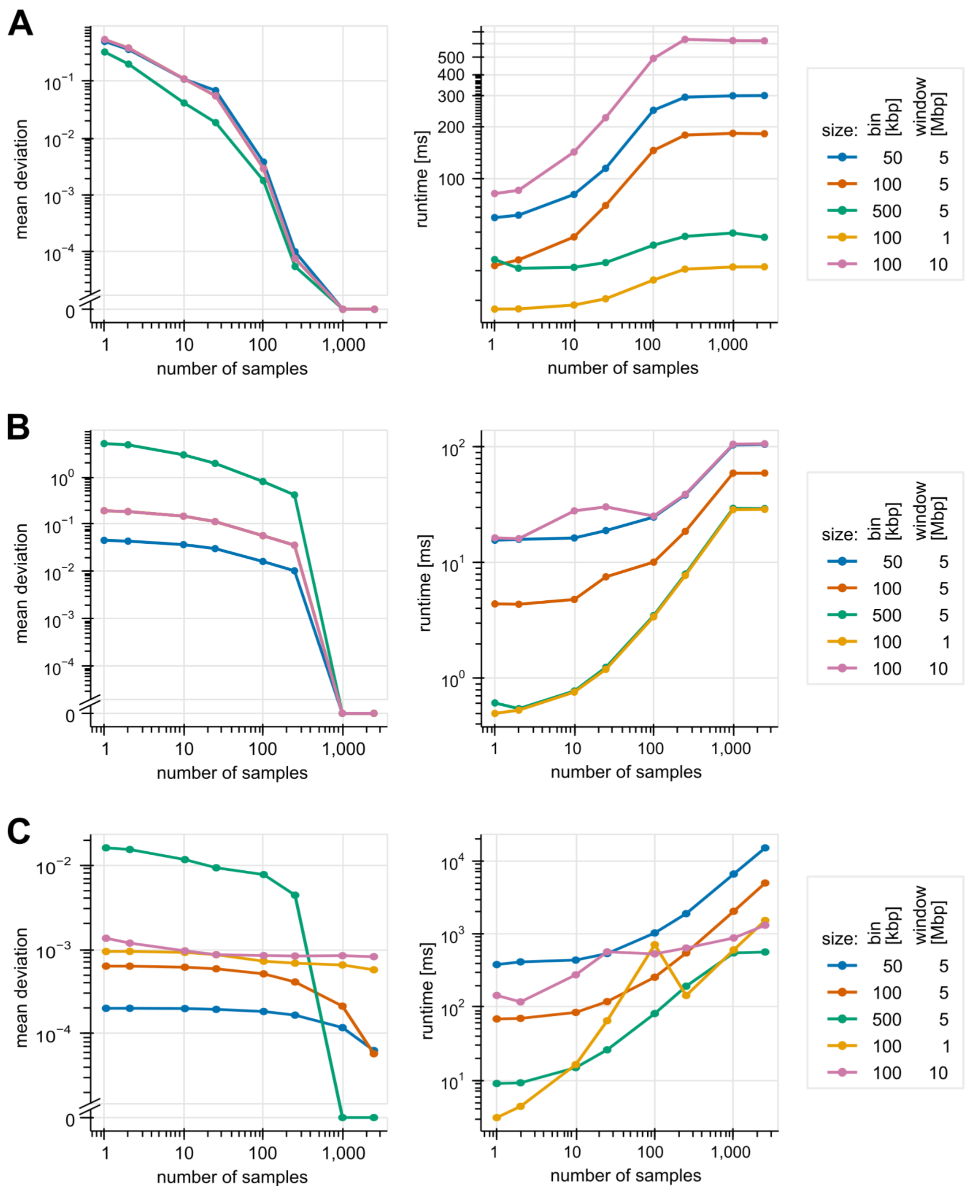

Supplementary Figure 13. Extended version of Figure 3B for (**A**) DDD, (**B**) Associated Slices, and (**C**) Binomial test normalization.

Supplementary Note 10 – Extended benchmarking

Below we show that neither the active filters nor genome size significantly affect the time required to compute one heatmap. While a larger number of active filters slightly increases runtime, no clear trend can be observed for genome size.

Next, we investigate index build times for various active filters, different genome sizes, and number of interactions. We find that index build times rise with increasing amounts of data, be it number of filters, genome size, or number of interactions. Compared to the index size benchmarking in Figure 3 A and C of the main text, we observe that build time rises linearly with both the number of interactions and

genome size, whereas index size rises sub-linearly with the number of interactions. Lower base resolutions reduce index build times.

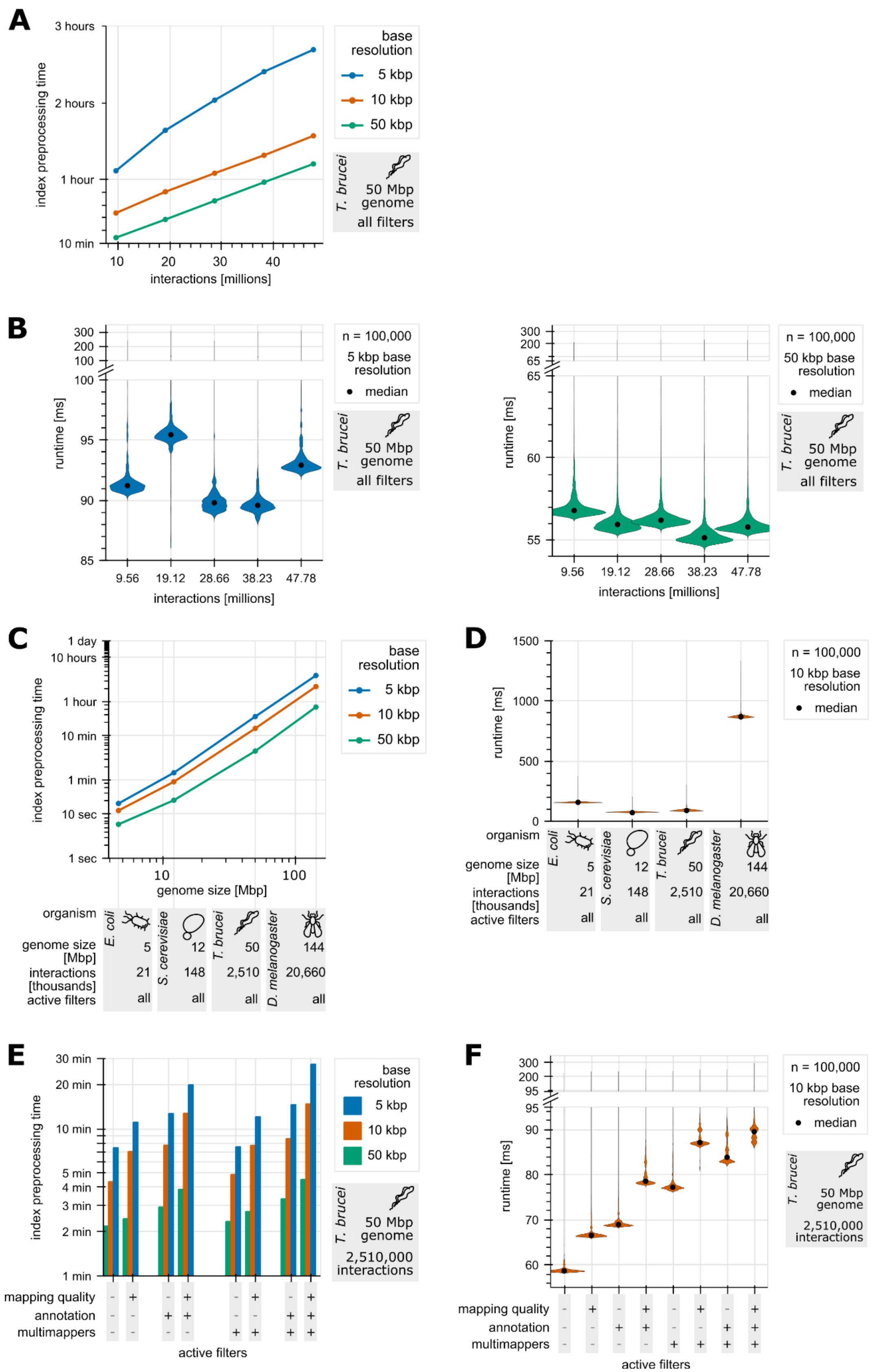

(Figure legend on next page.)

Supplementary Figure 14. Extended benchmarking of index size and speed. (**A**) Time required to preprocess one index as a function of the number of interactions. (**B**) Time required to compute one heatmap using default parameters for various base resolutions and number of unique interactions. (**C**) Time required to preprocess one index as a function of genome size. (**D**) Time required to compute one heatmap using default parameters for various genome sizes. (**E**) Time required to preprocess one index for various combinations of active filters. (**F**) Time required to compute one heatmap using default parameters for various combinations of active filters.

Supplementary Note 11 – Number of dimensions occupied by each filter

Here, we provide a table of the number of dimensions (i.e. the amount of resources) occupied by each filter. See Supplementary Figure 14E & F in Supplementary Note 10 and Figure 4D of the main text for an analysis of the filters impact on runtime speed, index size, and index buld time.

Filter	Occupied dimensions in the index	Dimension(s) inverted?	Other impacts?
(mapping loci of interactions)	dimensions 1 and 2	no	no
mapping quality	dimension 3	yes	no
multimappers	dimensions 4 and 5	no	yes: since we need to store rectangles instead of points now, each datapoint requires four prefix sum entries instead of one. See the methods section for more details.
annotation	dimension 6 and upwards; two dimensions are required per annotation type; by default we only process the gene annotation.	no	no

Supplementary Table 1. Overview of the number of dimensions used by each filter.

Supplementary Note 12 – Prefix sums require more datapoints than count matrices

Below we show an example for a set of interactions, its count matrix, and prefix sum matrix. For the count matrix, non-zero entries occur merely in places where at least one interaction exists. In a compressed matrix, merely those entries would need to be stored. With prefix sums, entries are continuously increasing. However, a compression merely needs to store entries where the stored value changes. The values of empty fields can then be inferred from the last stored entry. Such a change of a stored value occurs for all positions with interactions. However, such changes occur for positions, where

the columns and rows of interactions intersect (squares with red background). Due to these additional locations, any compression for prefix sums needs to store more data than a compression for count matrices.

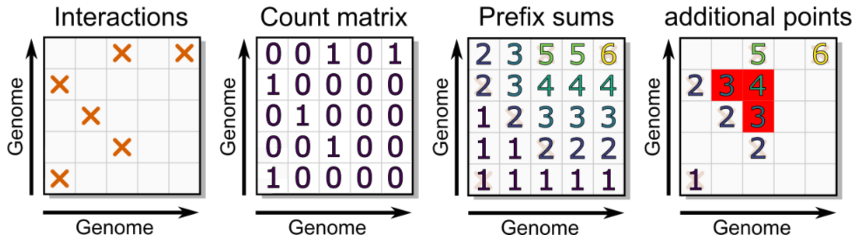

Supplementary Figure 15. The count matrix (2nd column) and prefix sums (3rd column) for a set of interactions (1st column). The final column shows the positions where prefix sums change but the count matrix is zero (highlighted in red).

References

1. Li, X. *et al.* GRID-seq reveals the global RNA–chromatin interactome. *Nat Biotechnol* **35**, 940–950 (2017).
